# Predicting the distribution of ground fissures and water-conducted fissures induced by coal mining: a case study

**DOI:** 10.1186/s40064-016-2609-3

**Published:** 2016-07-04

**Authors:** Kunyang Zhao, Nengxiong Xu, Gang Mei, Hong Tian

**Affiliations:** School of Engineering and Technology, China University of Geosciences, No. 29 Xueyuan Road, Beijing, 100083 China; Faculty of Engineering, China University of Geosciences, No. 388 Lumo Road, Wuhan, 430074 China

**Keywords:** Numerical simulation, Mining subsidence, Ground fissures, Water-conducted fissures, Tensile deformation

## Abstract

**Introduction:**

The use of Top Coal Caving for exploiting the thick coal seam with shallow buried depth most likely has a strong negative impact on the stability.

**Case description:**

Anjialing No. 1 Underground Mine is located in Shuozhou City, Shanxi Province of China. The 4# Coal Seam of this coal mine is the thick coal seam with shallow buried depth, which has the thickness of 12 m and the depth of 180 m in average. This paper focuses on predicting the distribution of ground fissures and water-conducted fissures induced by the exploiting of the 4# Coal Seam.

**Discussion and evaluation:**

We first create a 3D computational model, and then use FLAC$^{3D}$ software to simulate the mining of coal seam. We then calculate the displacements and tensile strain of the ground surface and strata, and predict the distribution of the ground fissures and water-conducted fissures. Finally, we further analyze the possibility of the perviousness and air leakage of the coal mine on the basis of the predicted distribution of fissures.

**Conclusions:**

The prediction results indicate that: (1) the water-conducted fissures are strongly developed and go through the Neogene aquifuge in some region; thus, it may lead to potential perviousness of coal mine; (2) part of these water-conducted fissures connect with the ground fissures; and this behavior may cause the risk of air leakage.

## Background

The use of Top Coal Caving for exploiting the thick coal seam with shallow buried depth typically has a strong impact on the stability, and most likely causes serious damage to the overlying strata. The serious damage to the strata overlying the mined-out area may further leads to the occurrence of water-conducted fissures and ground fissures.

If the water-conducted fissures completely go through the overlying aquifuge, then the water contained in the aquifer will most likely flow into the mined-out area. Furthermore, this will increase the inflow, and even lead the roof water inrush. In addition, if the water-conducted fissures connect with the ground fissures, then the leakage channel will be formed, which probably would destroy the underground ventilation cycle, and may lead to spontaneous combustion of coal and other dangers. Therefore, the prediction of the distribution of the ground fissures and water-conducted fissures induced by the mining subsidence is needed to be carried out to guarantee the safety in mine production.

Ground fissures and water-conducted fissures are caused by the deformation and movement of the overlying strata during coal excavation. The most important issue in predicting the distribution of the ground fissures and water-conducted fissures is to simulate the overlying strata behavior when mining coal seam. To address the above problem, several efforts have been conducted by adopting the methods of field measurement (Li et al. 2004; Xu et al. 2012; Zhao et al. 2013, similar physical simulation (Yanli et al. 2011; Miao et al. 2011; Ju and Xu 2013; Wang et al. 2015a; Ju and Xu 2015), and numerical simulation (Yoo and Lee 2008; Islam et al. 2009; Singh and Singh 2009; Tan et al. 2010; Zhang et al. 2013; Gao et al. 2014; Wang et al. 2015b). More specifically, based on GPS monitoring results, Zhao et al. (2013) analyzed the characteristics of ground deformation and ground fissures, as well as discussed their mechanisms and development trends. Xu et al. (2012) studied the mining-induced deformation of overlying strata and the time–space evolution law of fissures by the methods of field measurement and physical simulation. Wang et al. (2015a) conducted physical simulation experiments to study and distribution of overburden fissures induced by mining. According to the practical conditions, Tan et al. (2010) and Zhang et al. (2013) thought the discrete element method is an effective way to simulate the process of mining coal seam and studied the overburden fracture evolution laws. Islam et al. (2009) used the finite element method and boundary element method to established two models and evaluated overlying strata failure and fracture heights that resulted from these coal extraction operations.

Among those methods, the numerical simulation is one of the most practical and effective methods, especially the one that takes advantage of the Finite Difference Method (FDM) based software FLAC and ${\hbox {FLAC}}^{3D}$ (Alejano et al. 1999; Xie et al. 1999; Yasitli and Unver 2005; Unver and Yasitli 2006; Venticinque et al. 2014). For example, Jeromel et al. (2010) analyzed the geomechanical processes during sub-level coal excavation by using ${\hbox {FLAC}}^{3D}$. They found that the modeling of caving process by using FLAC$^{3D}$ to simulate the advancing of longwall coal mining was practical. Xu et al. (2013) used ${\hbox {FLAC}}^{3D}$ to conduct a numerical simulation of overlying strata movement caused by coal mining and predicted mining-induced surface subsidence.

Anjialing No.1 Underground Mine is located in Shuozhou City, Shanxi Province, the People’s Republic of China. In the coal mining area, the thickness of the 4# Coal Seam is about 12 m in average; and the depth is about 100–300 m. When using the Top Coal Caving to exploit the 4# Coal Seam, it typically has a strong impact on the stability, and most likely induces serious damage to the overlying strata. The serious damage to the strata overlying the mined-out area may further leads to the occurrence of water-conducted fissures and ground fissures.

This paper presents a case study on predicting the distribution of ground fissures and water-conducted fissures induced by the exploiting of the 4# Coal Seam of Anjialing No.1 Underground Mine at Panel 4106. We first create a 3D computational model according to the geological background in the study area. We then use the computer program ${\hbox {FLAC}}^{3D}$ 4.0 to simulate the mining of coal seam. We calculate the displacements and tensile strain of the ground surface and rock strata, and predict the distribution of the ground fissures and water-conducted fissures according to the distribution of the above displacements and tensile strain. Finally, we further analyze the possibility of the perviousness and air leakage of the coal mine on the basis of the predicted distribution of fissures.

## Geological background and mining history

### Geological background

#### Strata

**Fig. 1:**
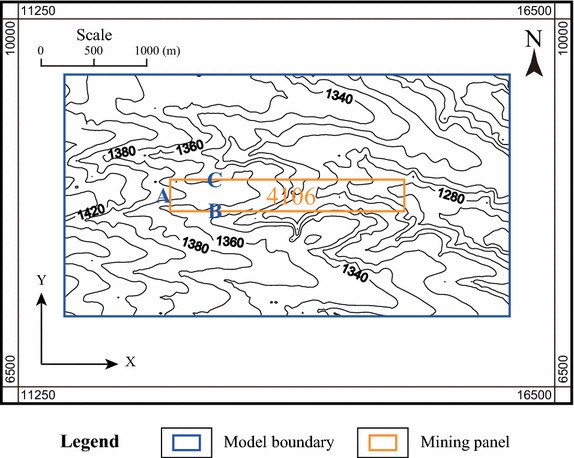
The contour map of the ground surface in the study area

In the region where Panel 4106 locates, the ridges and valleys stagger from the south to the north. And the valleys are developed in the “V-shape” with the erosion depth of 40–70 m; see Fig. 1.

It has been revealed through drilling that the stratigraphic sequence in this area, in the order from oldest to newest, is Ordovician, Carboniferous, Permian, Neogene and Quaternary. More details are listed as follows.

1. The Ordovician (O) stratum is composed of gray or dark gray thick-bedded crystalline limestone, which is pure in quality and high in strength. The average thickness of the Ordovician stratum is about 400 m.
2. The Carboniferous (C) stratum is divided into the following two formations, from the bottom to the top.
  The Benxi formation ($\hbox {C} _{2}$b), which has the average thickness of about 38.53 m, is composed of gray mudstone, sandy mudstone, and siltstone. The contact relationship between this formation and the underlying Ordovician stratum is a parallel unconformity.
  The Taiyuan formation ($\hbox {C} _{3}$t), which has the average thickness of about 79.76 m, is the primary coal-bearing stratum; and it is composed of gray sandstone, dark gray sandy mudstone and coal seam. The contact relationship with the underlying stratum is a conformity.
3. The Permian (P) stratum is divided into the following three formations, from the bottom to the top.
  The Shanxi formation ($\hbox {P} _{1}$s) is composed of medium-coarse quartzose sandstone, gray–black sandy mudstone, and siltstone. The average thickness of the Shanxi formation is about 59.61 m.
  The Xiashihezi formation ($\hbox {P} _{1}$x) is mainly composed of three parts. The bottom part is conglomeratic coarse sandstone and the middle part is brown and yellow–green coarse sandstone. The upper is the interbed of fine sandstone and siltstone interlaid with clay. The average thickness of the Xiashihezi formation is about 49.57 m.
  The Shangshihezi formation ($\hbox {P} _{2}$s) is mainly composed of gray–green, purple sandy mudstone, siltstone, and gray–green conglomeratic coarse sandstone. The average thickness of the this formation is about 25 m.
4. The Neogene (N) stratum is composed of brownish red clay and mild clay containing iron manganese spots, and its contact with the Permian is an unconformity. The average thickness of the Neogene stratum is about 23.74 m.
5. The Quaternary (Q) stratum is composed of yellowish brown sandy loam, mild clay, sandy loam soil, etc. and its contact with the Neogene is an unconformity. The average thickness of the Quaternary stratum is about 25.96 m.

#### Aquifer and aquifuge

The main overlying aquifer is derived from the local Quaternary aquifer. The pore water containing in the Quaternary aquifer is relatively weak and in general recharged mainly by atmospheric water and surface water. The overlying aquifuge locating in the Neogene stratum, is mainly composed of brown–red clay and mild clay which are widely distributed and have excellent impermeability. As listed in Table 1, the average distance between aquifuge and the 4# Coal Seam roof is about 141.5 m.

### The methods and processes of mining

**Fig. 2:**
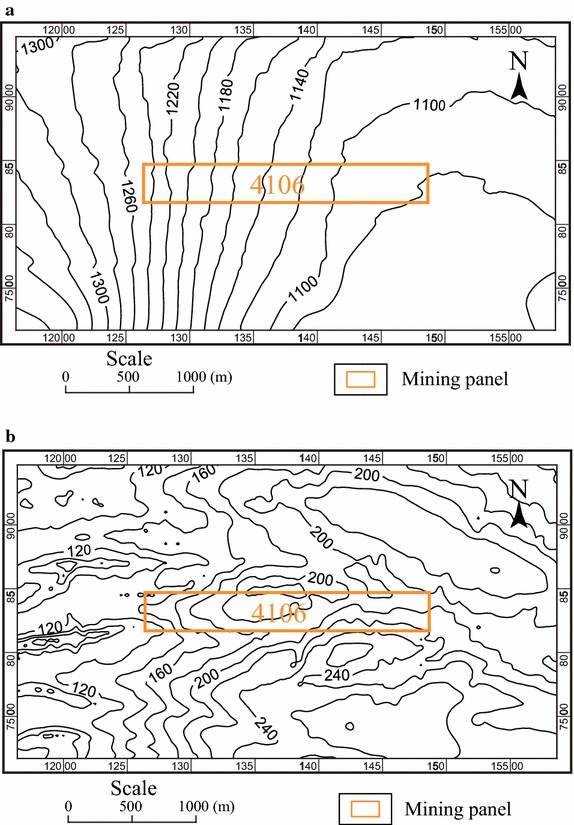
Elevation contours and depth contours of the coal seam roof. **a** Elevation contours; **b** depth contours

Panel 4106 is of 300 m width and 2226 m length; see Fig. 2. In the mining area, the average thickness of the 4# Coal Seam is 12 m; and the mining depth varies in the range of approximately 120–240 m. The top-coal-caving technique is adopted to extract the coal. The direction of mining is from west to east, with the speed of 200 m per month.

**Table 1:** The strata

Stratigraphic unit		Coal seam and layer	Floor depth (m)	Thickness (m)
System	Formation			
Quatemary		Aquifer	25.96	25.96
Neogene			42.38	16.42
		Aquifuge	49.70	7.32
Permian	$\hbox {P}_{2}$s		74.70	25.00
	$\hbox {P}_{1}$x		124.27	49.57
	$\hbox {P}_{1}$s		183.88	59.61
Carboniferous	$\hbox {C}_{3}$t	4# Coal Seam	195.88	12.00
			263.64	67.76
	$\hbox {C}_{2}$b		302.17	38.53
Ordovician			702.17	400.00

## Predicting the distribution of ground fissures and water-conducted fissures

### Overview of our method

To address the problem of whether the exploiting of Panel 4106 would lead the perviousness and air leakage of the mined out area, we adopt the numerical simulation method to predict the distribution of the ground fissures and the water-conducted fissures. Our prediction method is roughly composed of three steps.

- *Step 1* The creating of a 3D geological model
  According to the borehole logs, geological maps, and other stratigraphic data of the study area, we employ the mapping modeling approach to building the 3D geological model that can well reflect the actual stratigraphic layout (Xu and Tian 2009).
- *Step 2* The simulating of mining process
  We first determine the mining process of Panel 4106, and then use the software ${\hbox {FLAC}}^{3D}$ 4.0 to simulate the entire mining process, and calculate the distribution of the displacement and strain of the ground surface and overlying strata.
- *Step 3* The predicting of the distribution of fissures The horizontal tensile deformation of the ground surface and the overlying strata can heavily affect the size and scale of the fissures. Therefore, we predict the distribution of fissures by analyzing the tensile deformation of the ground surface and the overlying strata.

### Computational model

#### Model domain

**Fig. 3:**
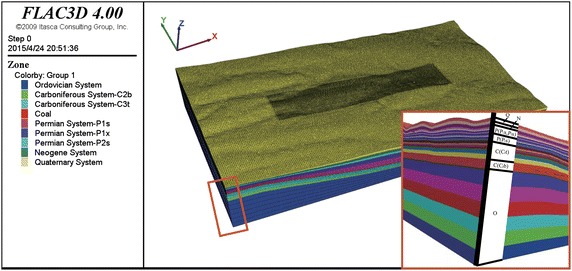
Computational mesh model

The domain of the computational model is determined according to the scale of the mining area of Panel 4106; see Fig. 1. For the sake of simplicity, we create a local coordinate system by setting the south–north direction illustrated in Fig. 1 as the Y-axis, the west–east direction as the X-axis, and the elevation direction as the Z-axis; see Fig. 3. The planar boundaries of the computational model are simply obtained by extending the boundaries of the mining area 1000 m along the west–east and the south–north directions, respectively. The distance along the west–east direction is 4226 m, while the distance along the south–north direction is 2300 m. The range of the elevation is between 812 and 1587 m. In planar view, the coordinates of the southwest and the northeast corners are (11,639, 7170) and (15,865, 9470), respectively.

#### Generalization of strata

The strata of the 3D geological model from top to bottom are as follows: Quaternary, Neogene, Permian, Carboniferous, and Ordovician. The geometric features of each stratum are determined by the borehole logs and geological maps.

#### Computational mesh model

The geological model was meshed using a set of triangular prism elements. A triangular prism has two triangular ends and three quadrangular sides. The triangular prism was selected instead of the more typical hexahedral mesh because the elevation of the strata in the Anjialing mine varies significantly in magnitude, and the nodes in a hexahedral mesh can give rise to relatively large displacements during the computing process. The displacements may result in extremely distorted elements and may cause computational problems. ${\hbox {FLAC}}^{3D}$ allows various options for shapes, including hexahedrons, tetrahedrons, pyramids, triangular prisms, etc. (Itasca Consulting Group 2009). Hexahedral elements are not used in this study, although they are normally the best choice for numerical simulation.

The presented computational mesh model is composed of 1,082,737 nodes and 2,052,648 elements. We create a gradient triangular mesh for the planar region/section using the following settings. The elements locating in and around the sub-region that represents the mining area are relatively small, which have the edge length of about 10 m. In contrast, those mesh elements that are far from the region representing the mining area are much bigger, which have the edge length of about 20 m. There are total 76,024 triangles in the planar section, and the planar triangular mesh is taken as the planar template mesh of each layer before interpolating. The planar triangular mesh consisting the above 76,024 triangles is then interpolated into corresponding surface triangular meshes according to the geometric features of 27 layers. Finally, the computational mesh model completely composed of prisms is created by connecting those corresponding triangles (i.e., where nodes of triangles are with the same *x, y* coordinates but different *z* coordinates) in each surface mesh; see Fig. 3.

#### The constitutive model and yield criterion

The Mohr–Coulomb yield criterion and the elastic-plastic constitutive model are used in this case study.

#### Boundary conditions

Displacement boundary conditions (Itasca Consulting Group 2009) are used in this study. The boundary conditions of the model are set as follows: (1) the fixed constraints in the X direction are applied the eastern and western boundaries of the model; (2) the fixed constraints in the Y-direction are applied the southern and northern boundaries; and (3) the fixed constraint in the Z-direction is applied at the bottom of the model. In addition, there are no constraints added to the upper surface of the model; see Fig. 3.

**Table 2:** The adopted physical–mechanical parameters

Strata	Rock formation	Elastic modulus (GPa)	Poisson’s ratio	Density (kN/$\hbox {m}^{3}$)	Friction angle ($^{\circ }$)	Cohesion (kPa)
O	–	17.80	0.12	2250	33	296.07
C	C$_{2}$b	14.84	0.13	2350	28	262.23
C	C$_{3}$t	14.10	0.14	2700	27	253.78
C	Coal	0.35	0.24	2350	22	70.00
P	P$_{1}$s	10.39	0.15	2650	26	203.02
P	P$_{1}$x	9.71	0.15	2517	27	194.57
P	P$_{2}$s	9.71	0.15	2517	27	194.57
N	–	3.11	0.17	2340	23	160.72
Q	–	0.05	0.26	1980	18	29.61

#### Calculation parameters

Table 2 lists the physical and mechanical parameters of the rock masses in the study area that were determined from physical and mechanical experiments on rocks and evaluations of the rock quality.

### The procedure of simulation

We use the software ${\hbox {FLAC}}^{3D}$4.0 to simulate the mining process. The simulation is carried out by 223 substeps, and in each step 10 m of the coal seam is planned to be exploited. In the simulating of each step of the mining process, the temporary stability in each step is desired to be obtained. After achieving the stability of the computational model, the calculation results such as the displacements of all nodes and the tensile strain of all elements are first recorded; and then the next step of mining process starts to be simulated.

### Surface movement

The rationality of three-dimensional calculation model and the adopted physical–mechanical parameters are verified as follow: if the simulated law of surface movement is in line with the actual situation, it can be explained that the 3D calculation model and the parameters are consistent with the actual results, and the results obtained are basically reliable.

**Fig. 4:**
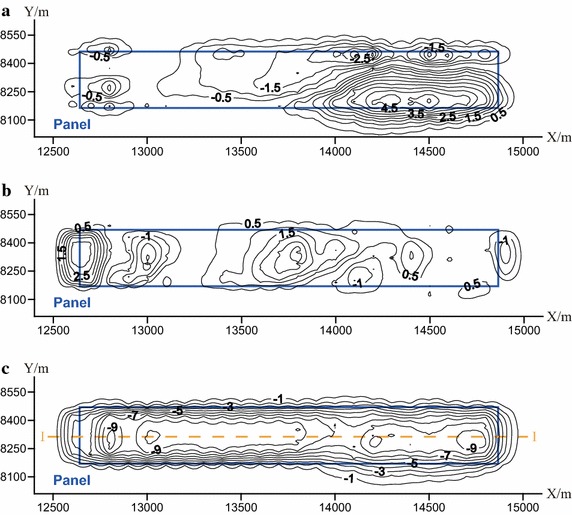
Contour maps of surface displacement at Panel 4106. **a** X direction; **b** Y direction; **c** Z direction

Figure 4a, b illustrates the contour map of the surface horizontal displacement after the completion of mining at Panel 4106. It can be observed that: (1) the contour of the horizontal displacement is approximately parallel to the boundaries of mining area; (2) the horizontal displacement above the center of the mined-out area is relatively small; (3) the greatest horizontal displacement arises on the surface region that is exactly above the boundaries of mining area. Due to the strong surface relief (i.e., the rise and fall ground surface), the displacement in the area above the mined-out area and the southern boundary of mining area is highly influenced by the rising ground surface. In general, the direction of the horizontal displacement orientates from the rising part of the ground surface to the fall part.

Figure 4c illustrates the contour map of the surface subsidence (i.e., vertical displacement) after the completion of mining at Panel 4106. It can be observed that: (1) the contour of the surface subsidence is approximately parallel to the boundaries of mining area; (2) the maximum surface subsidence (10.48 m) arises in the area above the center of the mined-out area; (3) the surface subsidence gradually decreases from the area above the center to the boundary of the mined-out area.

As can be observed in Fig. 4, the final subsidence basin can be roughly divided into three zones, including the central zone, the compressive zone, and the tensile zone. In the central zone, the subsidence is uniformly distributed, and can achieve the maximum value, while in contrast the horizontal displacement is quite small. The compressive zone is the region that is located between the boundary of the subsidence basin and the central zone, in which the subsidence is not uniformly distributed and compression arise due to the falling rock or soil body. The tensile zone is located on the boundary of the subsidence basin, in which the subsidence gradually reduced to zero; and tensile deformation always is produced due to the horizontal surface displacement that moves towards the center of the subsidence basin.

The surface movement results show that the final subsidence basin can be roughly divided into three subzones which are quite consistent with the theoretical distribution form, and the subsidence coefficient obtained from the maximum surface subsidence is consistent with the observed values of the region. Therefore, it can be claimed that: the computational model and the adopted parameters are validated.

### The predicting of ground fissures

**Fig. 5:**
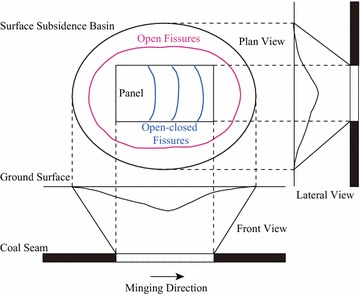
A simple illustration of ground fissures

The ground fissure can be roughly classified into two categories according to its distribution; see Fig. 5.

The first category is the open fissures which locate around the boundaries of the mining area and extend in the direction that is approximately parallel to the boundaries of mining area. The open fissures have a relatively large width and gap, and always exist in the process of mining. After finishing the entire mining process, the open fissures are oval-shaped distributed around the boundary of the panel. Even when the ground surface is stable, the open fissures in general will not become closed.

The other category is the open–closed fissures, which curvedly distribute directly above and in front of the working face. This type of ground fissures extends in the direction that is approximately perpendicular to the direction of the advancing working face. The width and gap of the open–closed fissures are relatively small, and the length is approximately the same as the width of the panel. In the process of mining, the open–closed fissures are first open and then gradually closed.

The major differences between the open and the open–closed fissures are: (1) the position of fissures’ distribution, (2) the form of fissures, and (3) whether the fissures will be closed after their production.

In general, the ground fissures would be formed for Quaternary loess layer when the tensile deformation reaches 1–5 mm/m. In this work, the region in which the tensile deformation of the ground surface exceeds 5 mm/m is considered as the area where the ground fissures would most likely be formed.

**Fig. 6:**
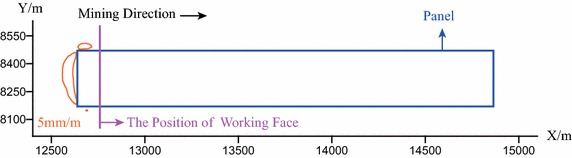
The contour map of the tensile deformation of the ground surface when the working face is extended to the position of 120 m

In the process of the first 11 steps of mining, i.e., when the working face is extended from the beginning to the length of 110 m, the calculation results indicates that the tensile deformation of the ground surface in the mining area is far less than 5 mm/m. This also suggests that: (1) in this process the impact of the underground mining on the ground surface is quite small; (2) the ground fissures cannot be formed. Figure 6 presents the contour map of surface tensile deformation when the length of the working face is extended to 120 m. It can be observed from the Fig. 6 that: the region where the tensile deformation is greater than 5 mm/m occurs around the boundaries of the mining area. It also means that the open fissures have occurred on the ground surface.

**Fig. 7:**
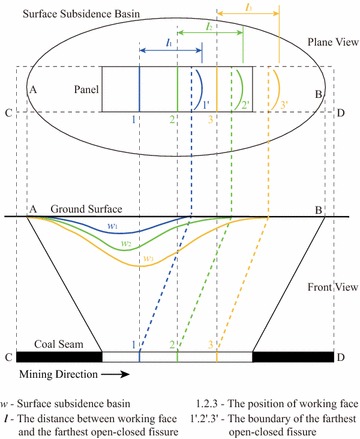
The distance between the advanced working face and the corresponding farthest open–closed fissure

For the sake of simplicity, the horizontal distance between the position of the current working face and the boundary of the open–closed fissures along the mining direction is denoted as ***l***; see Fig. 7. For example, when the working face is extended to the Position 1 and the farthest open–closed fissure is expanded to the Position 1′, then the distance between the Position 1 and Position 1′ is labeled as ${\varvec{l}}_{1}$; similarly, there exist the distances ${\varvec{l}} _{2}$and ${\varvec{l}}_{3}$.

A version of the distance ${\varvec{l}}$ can be calculated in each step of mining. And the distance ${\varvec{l}}$ can reflect the relative position of the formed open–closed fissure. Thus, the behavior of the forming of the open–closed fissures can be clearly drawn by analyzing the change of the ${\varvec{l}}$ values in the process of mining.

**Fig. 8:**
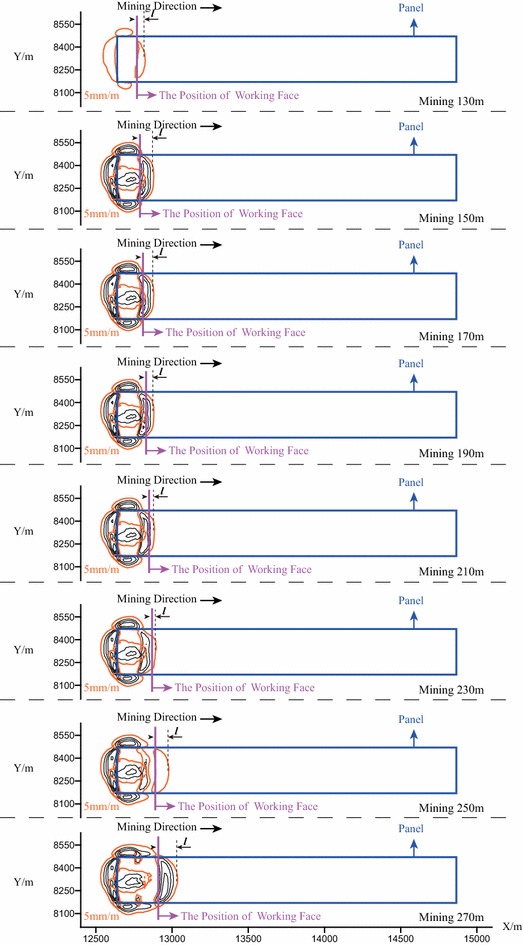
The contour map of the tensile deformation of the ground surface when the working face is extended from the position of 130 to 270 m (${\varvec{l}}$—the distance between the working face and the farthest open–closed fissure)

Figure 8 illustrates the contour maps of the tensile deformation when the working face is extended to the positions of 130, 150, 170, 190, 210, 230, 250, and 270 m. According to Fig. 8, it can be learned that when mining to the position of 130 m, the open–closed fissures begin to occur and the distance ${\varvec{l}}$ is quite small; with the advancing of mining, the open–closed fissure sustainably expand forward, and the distance ${\varvec{l}}$ gradually increases. When mining to the position of 150 m, the distance ${\varvec{l}}$ reaches the local maximum value.

In the subsequent steps of mining, the open–closed fissures no longer expand; and the ${\varvec{l}}$ gradually decreases. When mining further to the position of 250 m, the open–closed fissures re-expand forwards; and correspondingly the distance ${\varvec{l}}$ gradually increases. When mining to the position of 270 m, the distance ${\varvec{l}}$ reaches the local maximum value again. As can be seen from the above process, the open–closed fissures periodically expand along the direction that is perpendicular to the direction of mining.

**Fig. 9:**
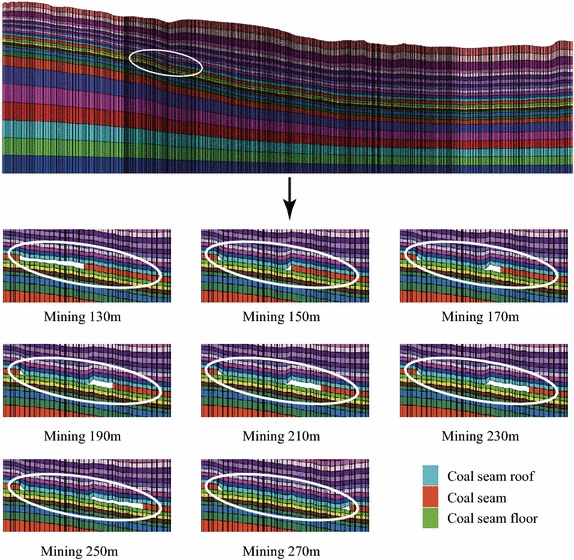
The illustration of the collapsing of coal seam roof on Section I–I when the working face is the extended from the position of 130 to 270 m

The forming of the surface fissures is strongly relevant to the collapsing of the coal seam roof. Figure 9 illustrates the collapsing of the coal seam roof when the working face is extended to the positions of 130, 150, 170, 190, 210, 230, 250, and 270 m. It can be observed from the Fig. 9 that: the seam roof began to bend down when mining to the position of 130 m. When mining to the position of 150 m, the seam roof collapsed for the first time. When further mining to the position of 250 m, the seam roof re-bended down again, while the seam roof collapsed for the second time when mining to the position of 270 m.

The above process indicates that: the coal seam roof does not collapse immediately after finishing the corresponding step of mining. When the mining length is relatively short, the seam roof bends down due to the impact of gravity.

When the advancing distance of the working face reaches a tertian limit, the seam roof begins to crack and collapse. And correspondingly, the collapsing of the seam roof would cause the subsidence of the ground surface. In addition, the tensile fissures occur in the boundary region of the subsidence basin, including (1) the open fissures that are located around the boundaries of the mining area and (2) the open–closed fissures that are distributed inside the mining area.

**Fig. 10:**
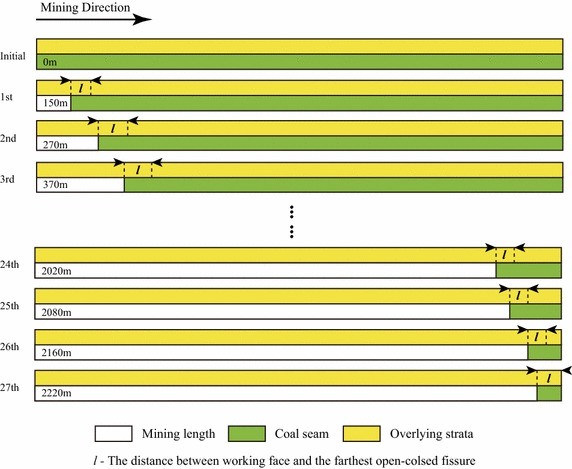
The periodical collapsing of the coal seam roof

Figure 10 illustrates the relationship of the mining length when the seam roof collapses and the horizontal distance between the boundary of the open–closed fissures and the position of the working face. In Fig. 10, it can be observed that the coal seam roof periodically collapse; and correspondingly the ground fissures periodically expand along the direction that is perpendicular to the direction of mining.

**Fig. 11:**
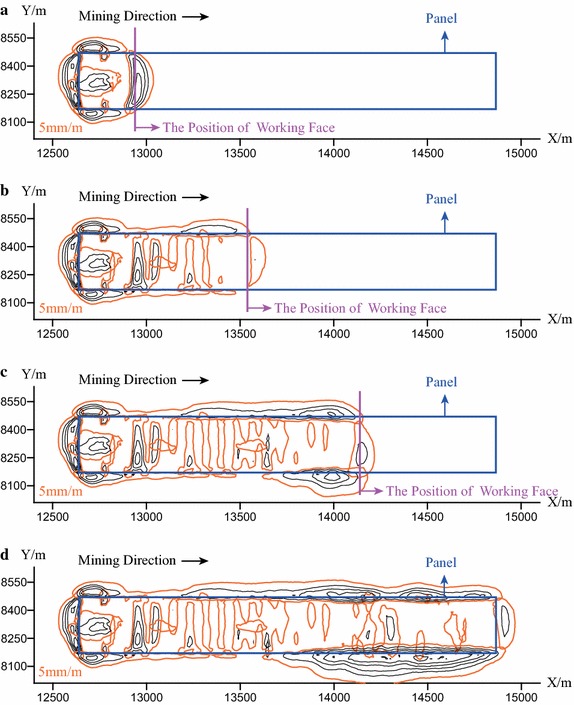
The contour map of the tensile deformation on the surface when the working face is advanced to the position of 300, 900, 1500, and 2226 m. **a** 300 m; **b** 900 m; **c** 1500 m; **d** 2226 m

Figure 11 describes the contour map of the tensile deformation of the ground surface when the working face is advanced to the position of 300, 900, 1500, and 2226 m. As can be seen in Fig. 11, with the advancing of the working face, the ground fissures expand along both the two directions that are parallel to and perpendicular to the direction of mining.

After finishing the entire mining process, the open fissures are oval-shaped distributed around the boundary of the working face, and gradually decrease along the direction that is perpendicular to the boundary of mining. In the mining area, some fissures expand along the direction that is perpendicular to the direction of mining. These fissures are derived from those incompletely closed fissures.

Moreover, after the completion of mining, the range of the contour of the tensile deformation around the south boundary of mining area is a little larger than that in the north side. This is because part of the ground surface locally rise near the south side of the boundary. Much greater tensile deformation is formed, which is the sum of the tensile deformation induced by the surface relief and the tensile deformation caused by the underground mining. Therefore, a large range of fissures occur in the south side of the boundary of the mining area.

### The predicting of water-conducted fissures

The water-conducted fissures are formed typically due to the tensile deformation of rock strata. In most cases, the nearer is the rock strata to the mined-out area, the stronger is the fracture of the rock strata. The forming and the distribution of the water-conducted fissures highly depend on the tensile deformation of the overlying strata induced by underground mining.

In general, the fissures would be formed for hard rock mass when the tensile deformation reaches 0–1 mm/m, while for weak rock mass the above limit of tensile deformation is about 1–2 mm/m. Due to the fact that the overlying strata in the study area is medium-hard, the value 2 mm/m is adopted as the threshold of tensile deformation to determine the position of the highest point of the water-conducted fissure.

**Fig. 12:**
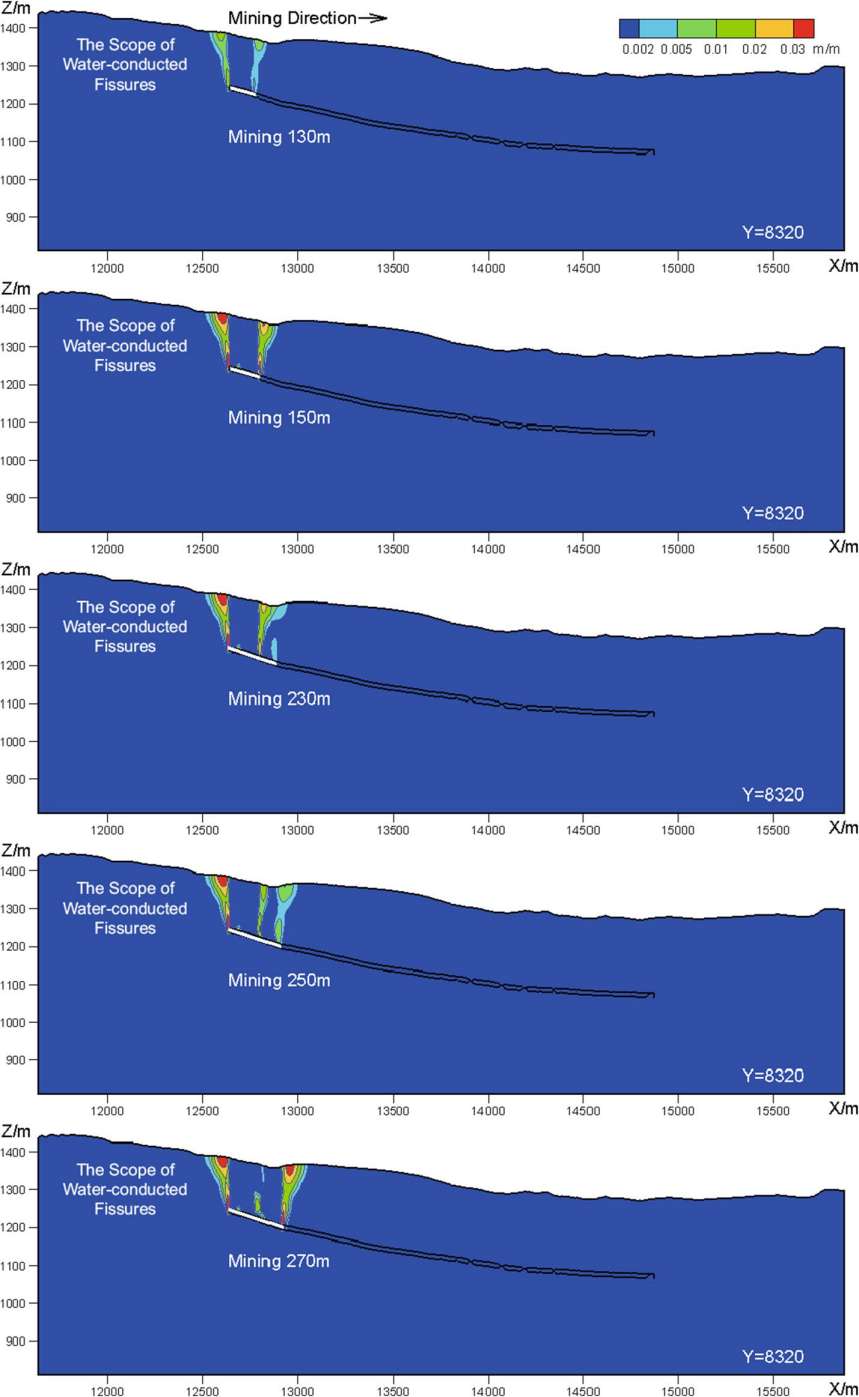
The contour map of tensile deformation on Section I–I (evolution laws of mining induced water-conducted fissures)

 Figure 12 provides the contour map of the tensile deformation on Section I–I in the process of mining. As can be seen in Fig. 12, when mining to the position of 130 m, the water-conducted fissures are first formed in the overlying strata locating above the boundary of the mining area; and when mining further, the water-conducted fissures gradually expand towards the ground surface; when mining to the position of 150 m, the coal seam roof collapses for the first time; and in this step, the water-conducted fissures in the overlying strata locating above the current position of the working face are fully developed.

With the advancing of mining, the range of the developed water-conducted fissures does not expand; see Fig. 12. When mining to the position of 250 m, the water-conducted fissures distributed above the mined-out area start to be closed; in addition, new water-conducted fissures are gradually formed in the overlying strata that locate in front of the position of the working face. The coal seam roof collapse for the second time when mining to the position of 270 m; and in this step the water-conducted fissures distributed in the overlying strata that locate in front of the current position of the working face are fully developed again.

The above described procedure indicates that the coal seam roof periodically collapses in the advancing of the working face; and the overlying strata periodically subside, the water-conducted fissures will also correspondingly be open or closed periodically.

**Fig. 13:**
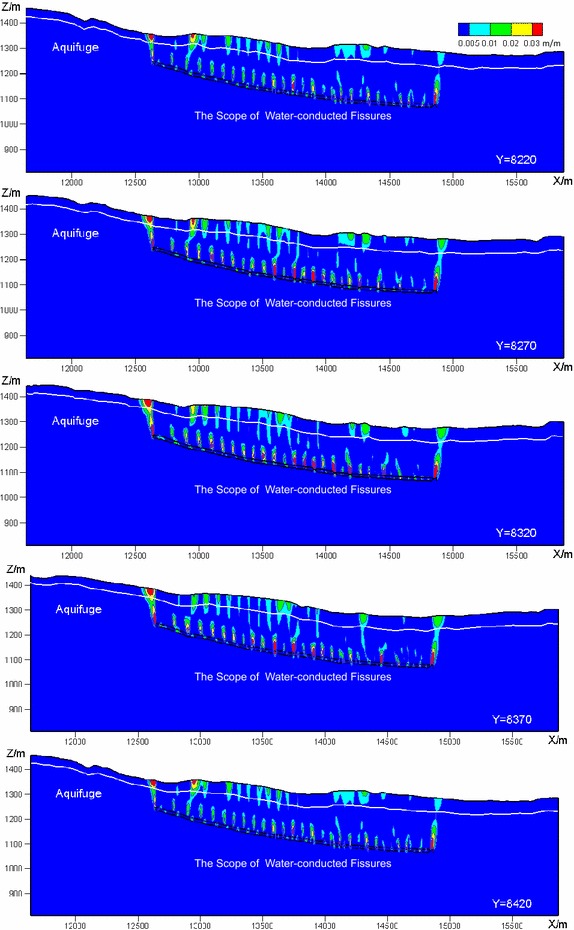
Contour map of the tensile deformation on typical cross-sections after the completion of mining (the final state of water-conducted fissures)

Figure 13 presents the contour map of the tensile deformation on five typical cross-sections after the completion of mining. It can be observed in Fig. 13 that: (1) the water-conducted fissures are distributed in the shape of saddle along the tilt direction of the working face; (2) most of the water-conducted fissures are distributed in the height of approximately 80 m, while the fissures are strongly developed in both the west and the east sides of the panel as well as partial area of the middle part, and go through the overlying strata in several local zones.

### Risk assessment

In the mining of the 4# Coal Seam, one may affect the safety of mining is the Quaternary aquifer. The underlying stratum of the Quaternary aquifer is the Neogene red clay, in which the water-conducted fissures cannot strongly develop. The above stratigraphic structure suggests that: the mine inflow in the process of mining is highly determined by the behavior whether the water-conducted fissures completely go through the Neogene aquifuge.

The average distance from the Neogene aquifuge to the coal seam is about 141.5 m. As can be seen in Fig. 13, the heights of most water-conducted fissures are about 80 m, which means the water-conducted fissures do not go through the overlying aquifuge, and will not bring the damage to the mining safety.

However, in both the west and the east sides of the panel, as well as partial area of the middle part, the water-conducted fissures are strongly developed with great height. This also means the water-conducted fissures completely go through the Neogene aquifuge, and connect to the Quaternary aquifer. Therefore, in this case, the ground water can infiltrate into the mine via the water-conducted fissures. In addition, part of these water-conducted fissures connects to the ground fissures. The air pressure along the water-conducted fissures will probably cause the air leakage, and bring potential damage to the mining safety.

## Discussion

### Surface subsidence

According to the behavior of the movement of ground surface, the subsidence basin is formed in the advancing of the working face. When mining to a certain distance, the surface movement is then impacted by the underground mining; and the subsidence basin can be formed. Moreover, the range of the subsidence basin gradually expands with the advancing of the working face. After the completion of the mining, the final subsidence basin can be roughly divided into three subzones, i.e., the central zone, the compressive zone, and the tensile zone.

1 $$\begin{aligned} q=\frac{W_{cm} }{M\cdot \cos \alpha } \end{aligned}$$

where *Wcm* is the maximum value of subsidence, *M* is the mining thickness, *q* is the surface subsidence coefficient and $\alpha$ is the angle of coal seam dip.

After finishing the mining, we have observed that: the maximum surface subsidence is about 10.48 m, and the subsidence coefficient is about 0.87 (Eq. 1). Compared to those subsidence coefficients obtained at several neighboring mines in Shanxi Province , the subsidence coefficient obtained at the Anjialing Mine is reasonable. More specifically, the subsidence coefficients at Xishan Mine, and Yangquan Mine, are $0.8\pm 0.1$ (Xiqu Coal Mine 12208 and 12209: 0.78; Xiqu Coal Mine 22101: 0.79; Xiqu Coal Mine 22108: 0.80; Zhenchengdi Coal Mine 12101: 0.85; Ximing Coal Mine 32903: 0.80) and 0.83 (No. 1 Coal Mine: 0.76; No. 2 Coal Mine: 0.90; No. 3 Coal Mine: 0.83; No. 4 Coal Mine: 0.83) (National Bureau of Coal Industry 2000), respectively.

It is clear that there is no significant difference among those subsidence coefficients; and this also suggests that the calculated subsidence coefficient in Anjialing Mine, i.e., 0.87, is reasonable.

### Ground fissures

According to the distribution of the ground fissures, we found that: (1) in the beginning of the mining process, the impact of underground mining on the deformation of ground surface is quite little, and cannot lead to the forming of ground fissures; (2) the ground fissures begin to occur when mining to the position of 120 m; and in the subsequent steps of mining, the ground fissures periodically expand along both the two directions that are parallel to and perpendicular to the direction of mining; (3) finally the open fissures are oval-shaped distributed around the boundary of the mining area, while the open–closed fissures are mainly distributed above the mined-out area.

**Fig. 14:**
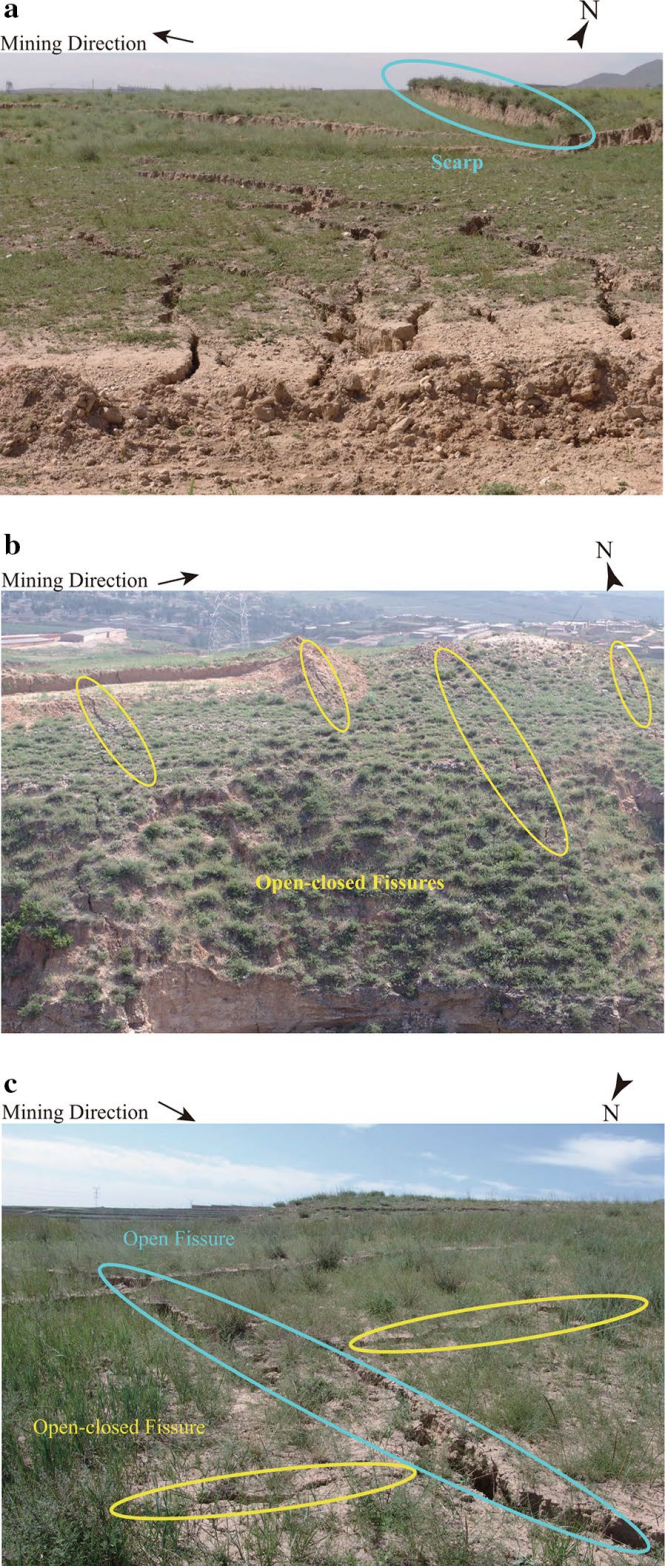
The photographs of the ground fissures when mining to the position of 300 m at Panel 4106 (locations A, B, C correspond to those three locations in Fig. 1). **a** Location A; **b** Location B; **c** Location C

Figure 14 presents the photographs of the ground fissures when Panel 4106 has been exploited 300 m. As can be seen from the photographs and Fig. 1, the Point A is the place for starting the mining process; and there exist scarps above the boundary of the mining area; and near the Point B, there are some open–closed fissures distributing along the direction that is perpendicular to the mining direction; furthermore, some criss-cross ground fissures occur at the Point C, including the staggered open fissures and open–closed fissures. The distribution of the above described open fissure and open–closed fissure is quite similar to the predicted distribution presented in this work. This also suggests that the simulating and assessing of the impact of underground mining on the movement and deformation of ground surface is practical.

To simulate the development of the ground fissures when exploiting the thick coal seam with shallow buried depth, Fan et al. (2011) used physical simulation to analyze the dynamic propagation and distribution of ground fissures that caused by longwall mining in shallow coal seams. Their results show that: (1) the ground fissures expand along the two directions that are parallel to and perpendicular to the direction of mining; (2) open fissures are oval-shaped distributed around the boundary of the mining area, while open–closed fissures are mainly distributed above the mined-out area; (3) in the process of mining, open fissures develop along the mining direction, and open–closed fissures are gradually closed and a new open–closed fissure appears in front of the working face. Wang et al. (2015b) conducted real-time monitoring on the surface fissures during the mining of the shallow depth seam. They thought the development of surface fissures generally goes through the following four stages: (1) surface fissure generation stage; (2) fissure expansion stage; (3) surface step subsidence stage; and (4) surface fissure closure stage.

The above comparisons indicate that: the numerical simulation results performed in the work is consistent with the observed distribution and other research work; thus the prediction method is effective and practical. The presented prediction method is capable of predicting the distribution of the ground fissures.

### Water-conducted fissures

According to the distribution of the water-conducted fissures, we observed that the water-conducted fissures were formed in the process when the overlying strata crack and collapse, and alter periodically from being open to being closed. After the completion of mining, the distributions of the water-conducted fissures are saddle-shaped, which is quite consistent with the theoretical distribution form.

By using the formula (Eq. 2) to calculate the maximum height of the water-conducted fissures (National Bureau of Coal Industry 2000), the calculated maximum height is about 79.3 m, which is also quite consistent with the numerical simulation results.

2 $$\begin{aligned} H_{li} =20\times \sqrt{\sum M } +10=79.3\,\hbox {m}, \end{aligned}$$

where $H_{li}$ denotes the maximum height of the water-conducted fissures (m); and $\Sigma M$ represents the accumulated thickness, which is set to 12 m in this work.

To simulate the development of the water-conducted zone when exploiting the thick coal seam with shallow buried depth, Zhang et al. (2013) used UDEC to analyze the evolution laws of overburden fractures in the mining process of the Ningwu Coalfield 4# Coal Seam in Panel 14101. Their numerical results indicated that fractures in the overburden strata dynamically varied with the working face advancing. The fracture zones extended in height and breadth, but closed at some local region at the same time. Wang et al. (2013) used the physical modeling experiment to simulate the distribution of overburden fractures induced by ascending mining and reveal the fracture evolution law. The results showed that: (1) the overburden failure development is trapezoidal, accompanied with the collapse in layer group and the developing height of fractures discontinuously jumps; (2) the water-conducted fissures experience a dynamic process from open to close.

The above comparisons indicate that: the numerical simulation results performed in the work is consistent with the empirical results and other research work; thus, the prediction method is effective and practical. The presented prediction method is capable of predicting the distribution of the water-conducted fissures.

### Applicability of our method

Our method is applicable to the general cases when: (1) employing the method of Top Coal Caving to (2) exploit the thick coal seam with shallow buried depth. It should be noted that, the critical tensile deformations of the ground surface and the overlying strata need to be determined based on the site-specific conditions. Besides, the presented method, in particular, requires very accurate information about the geological and mining conditions of the study area and the mechanical parameters of the overlying strata.

In summary, when using the Top Coal Caving to exploit the thick coal seam with shallow buried depth, our method can be used in general cases to predict the distribution of the ground fissures and water-conducted fissures.

### Suggested solutions to the geohazards in the mining process

According to the prediction results of ground fissures and water-conducted fissures, in those regions on the ground where: (1) the wide ground fissures and (2) the high water-conducted fissures may develop, should be filled in time when observed (for example, by Bulldozer rolling) to avoid air leakage and water inrush.

## Conclusion

A case study on predicting the distribution of the ground fissures and water-conducted fissures induced by the coal mining has been presented in this paper. The simulating of Top Coal Caving at Panel 4106 in Anjialing No. 1 Underground Mine has been carried out first. By carefully analyzing the calculated movement and deformation of ground surface and strata, the distribution of ground fissures and water-conducted fissures has been predicted; and the risk of perviousness and air leakage has been assessed.

The prediction results indicate that: (1) the water-conducted fissures are strongly developed in both the west and the east sides of the panel as well as partial area of the middle part, and go through the Neogene aquifuge in some region; thus, it may lead to potential perviousness of coal mine; (2) part of these water-conducted fissures connect with the ground fissures; and this phenomenon may cause the risk of air leakage.

The comparison of existing research work and observations with the obtained prediction results suggests that: the presented method is effective to predict the movement and deformation of ground surface and strata induced by the underground mining.

## References

[CR1] Alejano LR, Ramirez Oyanguren P, Taboada J (1999). Fdm predictive methodology for subsidence due to flat and inclined coal seam mining. Int J Rock Mech Min Sci.

[CR2] Fan G, Zhang D, Ma L (2011). Overburden movement and fracture distribution induced by longwall mining of the shallow coal seam in the Shendong Coalfield. J China Univ Min Technol.

[CR3] Gao F, Stead D, Coggan J (2014). Evaluation of coal longwall caving characteristics using an innovative UDEC Trigon approach. Comput Geotech.

[CR4] Islam MR, Hayashi D, Kamruzzaman ABM (2009). Finite element modeling of stress distributions and problems for multi-slice longwall mining in Bangladesh, with special reference to the Barapukuria Coal Mine. Int J Coal Geol.

[CR5] Itasca Consulting Group (2009) FLAC3D fast Lagrangian analysis of continua in 3 dimensions, version 4.0

[CR6] Jeromel G, Medved M, Likar J (2010). An analysis of the geomechanical processes in coal mining using the velenje mining method. Acta Geotech Slov.

[CR7] Ju J, Xu J (2013). Structural characteristics of key strata and strata behaviour of a fully mechanized longwall face with 7.0m height chocks. Int J Rock Mech Min Sci.

[CR8] Ju J, Xu J (2015). Surface stepped subsidence related to top-coal caving longwall mining of extremely thick coal seam under shallow cover. Int J Rock Mech Min Sci.

[CR9] Li X, Wang SJ, Liu TY, Ma FS (2004). Engineering geology, ground surface movement and fissures induced by underground mining in the Jinchuan Nickel Mine. Eng Geol.

[CR10] Miao X, Cui X, Wang J, Xu J (2011). The height of fractured water-conducting zone in undermined rock strata. Eng Geol.

[CR11] National Bureau of Coal Industry (2000) Pillar design and mining regulations under buildings, water, rails and major roadways. China Coal Industry Publishing House, Beijing

[CR12] Singh GSP, Singh UK (2009). A numerical modeling approach for assessment of progressive caving of strata and performance of hydraulic powered support in longwall workings. Comput Geotech.

[CR13] Tan YL, Zhao TB, Xiao YX (2010). Researches on floor stratum fracturing induced by antiprocedure mining underneath close-distance goaf. J Min Sci.

[CR14] Unver B, Yasitli NE (2006). Modelling of strata movement with a special reference to caving mechanism in thick seam coal mining. Int J Coal Geol.

[CR15] Venticinque G, Nemcik J, Ren T (2014). A new fracture model for the prediction of longwall caving characteristics. Int J Min Sci Technol.

[CR16] Wang C, Zhang N, Han Y, Xiong Z, Qian D (2013). Experiment research on overburden mining-induced fracture evolution and its fractal characteristics in ascending mining. Arab J Geosci.

[CR18] Wang C, Zhang N, Han Y, Xiong Z, Qian D (2015). Experiment research on overburden mining-induced fracture evolution and its fractal characteristics in ascending mining. Arab J Geosci.

[CR17] Wang F, Zhang C, Zhang X, Song Q (2015). Overlying strata movement rules and safety mining technology for the shallow depth seam proximity beneath a room mining goaf. Int J Min Sci Technol.

[CR19] Xie H, Chen Z, Wang J (1999). Three-dimensional numerical analysis of deformation and failure during top coal caving. Int J Rock Mech Min Sci.

[CR22] Xu N, Tian H (2009). Wire frame: a reliable approach to build sealed engineering geological models. Comput Geosci.

[CR20] Xu X, Zhang N, Tian S (2012). Mining-induced movement properties and fissure time–space evolution law in overlying strata. Int J Min Sci Technol.

[CR21] Xu N, Kulatilake PHSW, Tian H, Wu X, Nan Y, Wei T (2013). Surface subsidence prediction for the wutong mine using a 3-d finite difference method. Comput Geotech.

[CR23] Yanli H, Jixiong Z, Baifu A, Qiang Z (2011). Overlying strata movement law in fully mechanized coal mining and backfilling longwall face by similar physical simulation. J Min Sci.

[CR24] Yasitli NE, Unver B (2005). 3d numerical modeling of longwall mining with top-coal caving. Int J Rock Mech Min Sci.

[CR25] Yoo C, Lee D (2008). Deep excavation-induced ground surface movement characteristics—a numerical investigation. Comput Geotech.

[CR26] Zhang Y, Tu S, Bai Q, Li J (2013). Overburden fracture evolution laws and water-controlling technologies in mining very thick coal seam under water-rich roof. Int J Min Sci Technol.

[CR27] Zhao H, Ma F, Zhang Y, Guo J (2013). Monitoring and mechanisms of ground deformation and ground fissures induced by cut-and-fill mining in the Jinchuan Mine 2, China. Environ Earth Sci.

